# MIDLIFE PHYSICAL ACTIVITY AND HEARING IMPAIRMENT IN LATE LIFE: ATHEROSCLEROSIS RISK IN COMMUNITIES (ARIC) STUDY

**DOI:** 10.1093/geroni/igac059.1162

**Published:** 2022-12-20

**Authors:** Pablo Martinez-Amezcua, Erin Dooley, Emmanuel Garcia Morales, Nicholas Reed, Frank Lin, Jennifer Schrack, Jennifer Deal, Priya Palta

**Affiliations:** Johns Hopkins Bloomberg School of Public Health, Baltimore, Maryland, United States; Epidemiology, Birmingham, Alabama, United States; Cochlear Center for Hearing and Public Health, Baltimore, Maryland, United States; Johns Hopkins Bloomberg School of Public Health, Baltimore, Maryland, United States; Johns Hopkins Cochlear Center for Hearing and Public Health, Baltimore, Maryland, United States; Johns Hopkins Bloomberg School of Public Health, Baltimore, Maryland, United States; Johns Hopkins University, Baltimore, Maryland, United States; General Medicine, New York City, New York, United States

## Abstract

**Background:**

Cardiovascular risk factors are associated with worse hearing, but the role of mid-life physical activity (PA) on hearing loss at older ages is yet to be investigated.

**Methods:**

Among 3,198 participants of the Atherosclerosis Risk in Communities study, we investigated the association between self-reported mid-life PA (meets PA recommendations [≥150 minutes of moderate-to-vigorous PA/week] vs. not) and hearing loss (audiometric battery [pure-tone and speech-in-noise]) at older ages. We estimated differences in hearing between those who met and did not meet PA recommendations at mid-life and at late life adjusting for demographics, medical conditions, and noise exposure.

**Results:**

43.3% participants met PA recommendations at mid-life. These participants, compared to those who did not meet recommendations, had lower better hearing by 1.51 (0.46, 2.55) decibels, and 0.37 (0.01, 0.74) more words identified in the speech-in-noise test.

**Conclusions:**

Meeting PA recommendations in mid-life was associated with better hearing at older ages.

